# Distinct pathways for genetic and epigenetic predisposition in familial and bilateral Wilms tumor

**DOI:** 10.1186/s13073-025-01482-0

**Published:** 2025-05-08

**Authors:** Jenny Wegert, Silke Appenzeller, Taryn D. Treger, Heike Streitenberger, Barbara Ziegler, Sabrina Bausenwein, Christian Vokuhl, Conor Parks, Eva Jüttner, Susanne Gramlich, Karen Ernestus, Steven W. Warman, Jörg Fuchs, Jochen Hubertus, Dietrich von Schweinitz, Birgit Fröhlich, Norbert Jorch, Ralf Knöfler, Carsten Friedrich, Selim Corbacioglu, Michael C. Frühwald, Arnulf Pekrun, Dominik T. Schneider, Jörg Faber, Jana Stursberg, Markus Metzler, Nils Welter, Kathy Pritchard-Jones, Norbert Graf, Rhoikos Furtwängler, Sam Behjati, Manfred Gessler

**Affiliations:** 1 Developmental Biochemistry, Theodor-Boveri-Institute/Biocenter, Julius-Maximilians-University Würzburg, Am Hubland, Würzburg, 97074 Germany; 2 Comprehensive Cancer Center Mainfranken, University Hospital of Würzburg, Würzburg, Germany; 3 Wellcome Sanger Institute, Hinxton, UK; 4 Department of Pediatrics, University of Cambridge, Cambridge, UK; 5 Cambridge University Hospitals NHS Foundation Trust, Cambridge, UK; 6 Section of Pediatric Pathology, Department of Pathology, University Hospital Bonn, Bonn, Germany; 7 Department of Pathology, Schleswig-Holstein University Hospital, Kiel, Germany; 8 Department of Pathology, University of Würzburg, Würzburg, Germany; 9 Clinic of Pediatric Surgery, Charité – University Hospital Berlin, Berlin, Germany; 10 Department of Pediatric Surgery and Pediatric Urology, University Children’s Hospital, Tuebingen, Germany; 11 Department of Pediatric Surgery, Marien Hospital Witten, Ruhr-University Bochum, Bochum, Germany; 12 Department of Pediatric Surgery, Dr. von Hauner Children’s Hospital, LMU University Hospital, Munich, Germany; 13 Department of Pediatric Oncology and Hematology, University of Münster, Münster, Germany; 14 Evangelisches Klinikum Bethel, Universitätsklinikum OWL, Bielefeld, Germany; 15 Department of Pediatric Hematology/Oncology, Medizinische Fakultät Carl Gustav Carus, Technische Universität Dresden, Dresden, Germany; 16 Department of Pediatrics and Pediatric Hematology/Oncology, University Children’s Hospital, Carl von Ossietzky University, Klinikum Oldenburg, Oldenburg, Germany; 17 Children’s Hospital Regensburg, University of Regensburg, Regensburg, Germany; 18 Swabian Children’s Cancer Center, Pediatrics and Adolescent Medicine, University Hospital Augsburg, Augsburg, Germany; 19 Pediatric Hematology and Oncology, Klinikum Bremen, Bremen, Germany; 20 Clinic of Pediatrics, University Witten/Herdecke, Klinikum Dortmund, Witten, Germany; 21 Department of Pediatric Hematology/Oncology, Center for Pediatric and Adolescent Medicine, University Medical Center, Johannes Gutenberg-University, Mainz, Germany; 22 Department of Pediatrics and Adolescent Medicine, Ulm University Medical Center, Ulm, Germany; 23 Department of Pediatrics and Adolescent Medicine, University of Erlangen-Nürnberg, Erlangen, Germany; 24 Department of Pediatric Hematology and Oncology, Saarland University Hospital, Homburg, Germany; 25 UCL Great Ormond Street Institute of Child Health, University College London, London, UK; 26 Division of Pediatric Hematology and Oncology, Department of Pediatrics, Inselspital University Hospital, University of Bern, Bern, Switzerland

**Keywords:** Wilms tumor, Nephroblastoma, Genomic imprinting, Hereditary cancer, Cancer predisposition, Pediatric cancer, Multicentric tumors, WT1, BWS

## Abstract

**Background:**

Genetic predisposition is particularly common in children with the kidney cancer, Wilms tumor. In 10% of these children, this manifests as a family history of Wilms tumor or bilateral disease. The frequency and spectrum of underlying changes have not been systematically investigated.

**Methods:**

We analyzed 129 children with suspected Wilms tumor predisposition, 20 familial cases, and 109 children with bilateral disease, enrolled over 30 years in the German SIOP93-01/GPOH and SIOP2001 studies. We used whole exome, whole genome, and targeted DNA sequencing, together with MLPA and targeted methylation assays on tumor, blood, and normal kidney to determine predisposing changes.

**Results:**

Predisposing variants were identified in 117/129 children, comprising DNA variants (57%) and epigenetic changes (34%). Most children had predisposition variants in genes previously implicated in Wilms tumor: most prominently *WT1* (*n* = 35) and less frequently *TRIM28*,* REST*,* DIS3L2*, *CTR9*, *DICER1*, *CDC73*, and *NONO*. Nine children carried germline mutations in cancer predisposition genes not considered Wilms tumor predisposition genes, such as *CHEK2*, *CDKN2A*, *BLM*, *BRCA2*, *STK11*, and *FMN2*.

Predisposition via epigenetic BWS-IC1 alterations occurred as early somatic events, reflected by partial (mosaic) loss of imprinting or loss of heterozygosity at the *IGF2/H19* locus in normal kidney or blood. These patients rarely had a clinical diagnosis of Beckwith-Wiedemann syndrome (BWS).

Especially *WT1-*driven tumors follow a stereotypical pathway of germline *WT1* mutations becoming homozygous in renal precursor lesions through 11p LOH, which concomitantly activates imprinted *IGF2* expression, with subsequent WNT pathway activation leading to tumor growth. There is a high rate of multicentric tumors, which may have previously been missed in unilateral tumors. While Wilms tumor predisposition genes relied on somatic inactivation of the second allele, this was different for general cancer predisposition genes. The latter cases were often associated with additional oncogenic alterations, similar to tumors with epigenetic predisposition.

**Conclusions:**

We identified two main mechanisms of Wilms tumor predisposition: either germline genetic alterations of Wilms tumor and, less frequently, general cancer genes; or postzygotic mosaic imprinting defects activating *IGF2*. These findings inform future genetic screening and risk assessment of affected children and lend support to liquid biopsy screening for enhanced therapeutic stratification.

**Supplementary Information:**

The online version contains supplementary material available at 10.1186/s13073-025-01482-0.

## Background

Wilms tumor (WT), or nephroblastoma, is the most common pediatric renal tumor, affecting 1 in 10,000 children, mostly before the age of 6 years. This embryonal malignancy is thought to result from aborted or misdirected development of the fetal kidney, which manifests itself in a diverse spectrum of histological appearances [1, 2]. There is limited correlation between histology and underlying genetic causes, with *WT1* mutations preferentially found in stromal type and *TRIM28* mutations in epithelial type tumors. Progression to anaplasia is mainly driven by somatic *TP53* mutation. In the majority of cases, however, there is no link between specific genetic drivers and stromal, epithelial, and blastemal contribution.

Although most cases of WT are sporadic, approximately 1–2% of patients have a family history of WT, and 7–8% are reported with bilateral disease [3–5]. Both are expected to carry a genetic predisposition, in accordance with the two-hit model, where the first mutation is present already in the germline or occurs early in embryonic development, while the second is acquired somatically at a later stage [6]. The recent analysis of a WT cohort from the Netherlands revealed a much higher rate of (epi)genetic predisposition in WT of 33%. This suggests that some of these predispositions must have been overlooked in prior studies [7].

WT predisposition can either be associated with different genetic syndromes or with predisposition genes without apparent syndromic features. Examples for the latter are germline variants of *TRIM28*, *REST*, *NYNRIN*, *CDC73*, or *FBXW7* that can convey a risk for WT, but do not seem to affect the development or function of other organs [8]. Deletion of chromosome 11p13 underlies WAGR syndrome (Wilms tumor, Aniridia, Genitourinary anomalies, Range of developmental delay) that includes WT development due to *WT1* inactivation [9, 10]. Other syndromes associated with an elevated WT risk include Denys-Drash syndrome (DDS, *WT1* point mutations) [11], and several overgrowth syndromes: Perlman (biallelic germline *DIS3L2* mutation) [12], Simpson-Golabi-Behmel (*GPC3*/*GPC4*), *PIK3CA*-related overgrowth spectrum (PROS), and Beckwith-Wiedemann syndrome (BWS) [13].

BWS is driven by epigenetic alterations of imprinting at chromosome 11p15.5, and these patients are at risk of developing embryonal tumors, including WT or hepatoblastoma [14, 15]. Furthermore, loss of imprinting (LOI) or loss of heterozygosity (LOH) overlapping the BWS imprinting center 1 (BWS-IC1), especially the *IGF2* locus, has been described as an epigenetic change in up to 80% of sporadic WT [16–19]. The net result of having paternal IC1 imprints on both chromosome 11p15 loci is an elevated expression of *IGF2*. Deregulated imprinting is the most frequent driver of WT formation but also a relevant predisposing factor. The contribution of various genetic and epigenetic changes to WT predisposition has not been fully resolved, however.

We characterized children with suspected WT predisposition based on familial or bilateral disease. Using data from a large clinical cohort spanning three decades, we identified the underlying genetic and epigenetic events, revealing new insights into predisposition mechanisms. This analysis revealed key insights into germline vs. somatic mosaic predisposition, the heterogeneity of multicentric tumors, and the stereotypic sequence of events in *WT1*-driven disease.

## Methods

### Sample collection

Tumor and control samples were obtained from the German SIOP93-01/GPOH (8 patients) and SIOP2001/GPOH (121 patients) studies (approved by the Ethikkommission der Ärztekammer des Saarlandes, reference numbers 23.4.93/Ls and 136/01 and 248/13). Informed consent had been obtained from all patients/parents. All samples were pseudonymized. Biobank operation was approved by the Ethikkommission of the University of Würzburg (reference 336/21_z-sc).

### Sample processing

All samples had been snap-frozen, shipped on dry ice from local hospitals, and stored at −80 °C. Sections of frozen tumor and normal kidney were hematoxylin/eosin-stained and inspected by a reference pathologist (C.V.) for tumor cell content, cellular composition, signs of nephrogenic rests, and anaplasia. Tumor and normal kidney DNA and RNA of frozen tissue were isolated in parallel with QIAamp Mini Allprep kit (QIAgen) from 5 to 10 10 µm cryosections. DNA of PBMCs (peripheral blood mononuclear cells) was isolated as described [20]. Whole exome sequencing was performed on 118 tumor, 17 adjacent kidney, and 61 blood samples. Whole genome analysis was done on 46 tumor, 15 kidney, and 26 blood samples. Only targeted analyses of *WT1*, *CTNNB1*, *AMER1*, or *TRIM28* were performed on 32 tumor, 4 kidney, and 17 blood samples. Copy number profiles were extracted from WGS and WES data and independently repeated on all tumor samples by MLPA. Methylation testing of the BWS-IC1/2 region was also performed on each of the tumor samples. For 18 cases where no frozen kidney tissue was available for methylation analysis, DNA was isolated from FFPE material with the QIAamp DNA FFPE Advanced kit.

### WES

Whole-exome sequencing (WES) was performed by Novogene (Cambridge, UK) using the Agilent SureSelect Human All Exon V6 Kit (Agilent Technologies, CA, USA) with paired-end sequencing (PE-150) to obtain an average of 40 million reads. Initial quality assessment was performed using FastQC-v0.11.5 [21]. Adapters and low-quality reads were trimmed using TrimGalore-v0.6.1 [22] powered by Cutadapt-v2.3 [23].

Trimmed reads were mapped to the human reference genome (hg19) using BWA-MEM-v0.7.12 [24]. Sorting and indexing were performed using Picard-v1.125 [25] and SAMtools/HTSlib-v1.3 [26]. Duplicate reads were marked with Picard. GATK-v4.0.11.0 and v3.5 [27] were used for base recalibration and coverage calculation.

Germline variants including substitutions and small indels were called using GATK4 HaplotypeCaller and Scalpel-v0.5.3 [28]. Somatic substitutions and indels were identified with GATK4-MuTect2 and VarScan2-v2.4.1 [29], and Scalpel. Variants were annotated with ANNOVAR [30] and visually examined using the IGV browser [31]. Variants were reported if they had an impact on the protein sequence or affected a splice site and are rare in the population (< 2% in 1000g2015aug_all, ExAC_nontcga_ALL, gnomAD_exome_ALL, and gnomAD_genome_ALL) and if the position is covered by at least 10 reads and the alternative allele is covered by at least 3 reads and comprised at least 5%. Additionally, Varscan2 was used to detect loss of heterozygosity and copy number variations (CNVs).

### WGS

DNA processing, sequencing, and variant calling for whole genome sequencing (WGS) were done as part of The Little Princess Trust Knowledge Bank of Wilms Tumour, as described in [32]. Germline variants were considered when not described as common SNPs in gnomAD or ExAc (frequency < 1%) and predicted to be deleterious by variant prediction tools (Provean, SIFT, Polyphen2). Regions of known WT genes were manually inspected with IGV or JBrowse for CNV or structural variants not detected by variant callers.

### Targeted analysis

Targeted Sanger sequencing of suspected genes was performed in 15 stromal (*WT1, CTNNB1*, and *AMER1*) and 5 epithelial (*TRIM28*) predominant tumors. Suspicious variants detected by WES or WGS were validated by targeted Sanger sequencing of the respective region in genomic DNA or cDNA. PCR was used to determine copy-neutral loss of heterozygosity (LOH) at 11p (markers *TH01*, *D11S1392*) or 3p (*D3S1358*). Primer sequences are given in Additional file 1: Table S1.

### MLPA

The WT-specific probemix P380 Wilms Tumour (MRC Holland) was used to determine copy number alterations at 1p, 1q, *MYCN*, *FBXW7*, *WT1*, 16p, 16q, *TP53*, and *AMER1* by MLPA (multiplex ligation-dependent probe amplification). The methylation status of BWS-IC1/IC2 on chromosome 11p15.5 was determined by methylation-sensitive MLPA ME030-BWS/RSS (MRC Holland). Data were analyzed using Coffalyzer.Net (MRC Holland); the percentage of affected cells was determined based on the mean of all probes in the respective region.

## Results

**Fig. 1:**
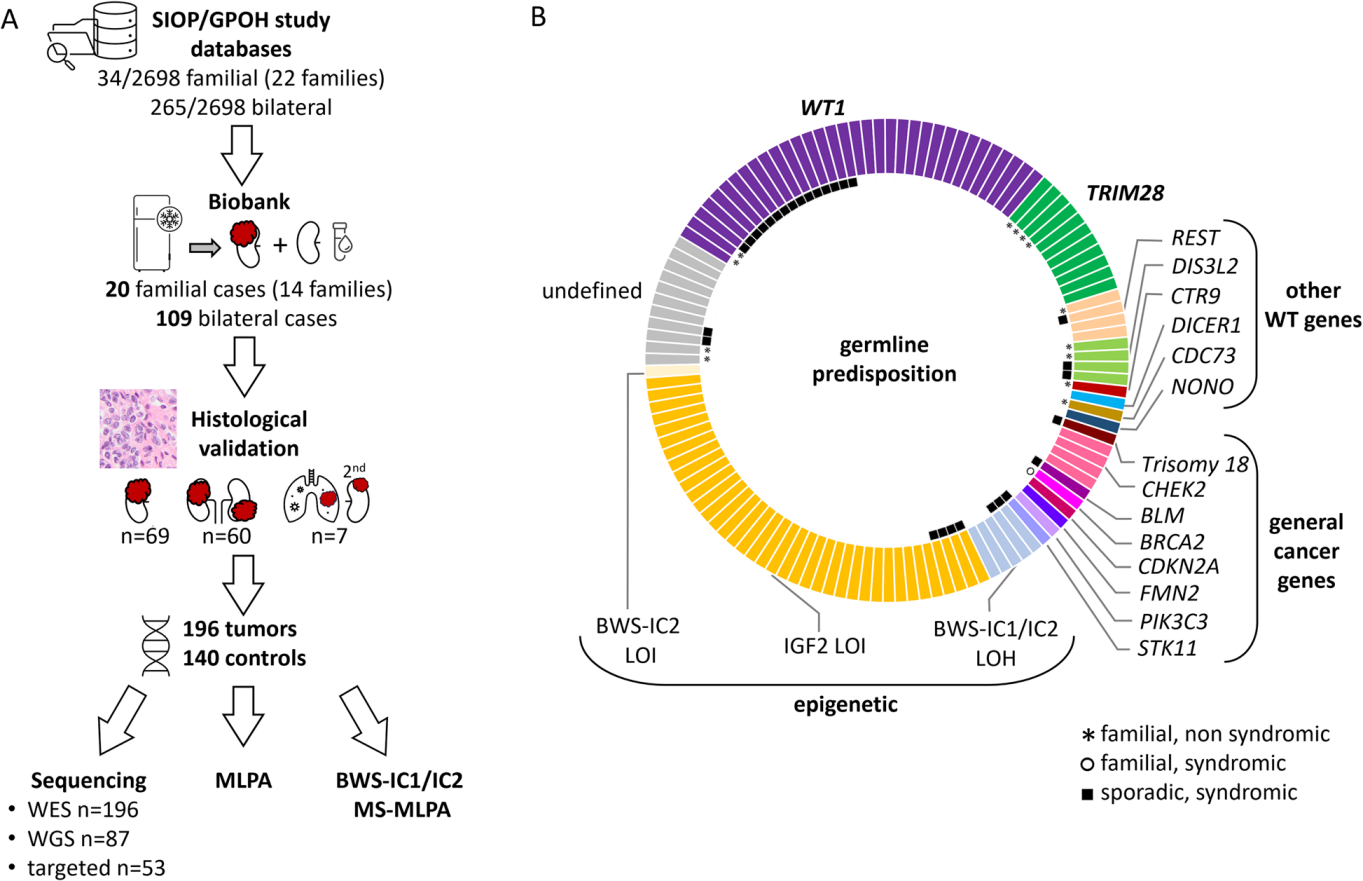
Sample characteristics and predisposing events in WT. **A** Patients with a family history of WT or bilateral disease were selected from the German SIOP93-01/GPOH and SIOP2001/GPOH studies. Sufficient tumor and control tissue was available for 129 WT patients. After histological validation of tumor and healthy kidney material, samples were subjected to MLPA for WT-specific copy number alterations and IGF2 imprinting status (BWS-ICR MS-MLPA). Tumor and control DNA was analyzed by either whole exome (WES), whole genome sequencing (WGS), or targeted Sanger sequencing of presumed driver genes. **B** Types of germline predisposition events. Only individual independent germline events are shown, i.e., a family with more than one patient analyzed is counted as one event. A total of 62 patients had germline mutations in known WT genes, while 11 patients had general cancer predisposing conditions, and 44 patients showed (mosaic) epigenetic events of the IGF2/H19 imprinting region

To elucidate genetic and epigenetic alterations that predispose to WT formation, we selected patients enrolled in the two consecutive German SIOP93-01/GPOH and 2001 studies who were clinically suspected of having a predisposition based on reported family history or bilateral disease. Among the 2698 patients registered between November 1994 and January 2022, there were 22 families (34 affected individuals) and 265 bilateral cases (Fig. 1A). These frequencies of 1.3% and 10% match literature values [3–5]. The term bilateral WT is used here to refer to disease affecting both kidneys with neoplasms, either WT and/or nephroblastomatosis. Nephroblastomatosis is a term used to describe kidneys exhibiting multiple or diffuse nephrogenic rests, putative precursors of WT.

Appropriate tissues (tumor, normal tissue) were available from 14 families with 20 WT patients. One family had three affected siblings, and in four families, material from two affected children was available. In addition, 109 patients with bilateral disease were included, with tumor material from both sides in 60 cases. We obtained concurrent or subsequent metastases from 7 patients, resulting in 196 tumor samples from 129 patients. An adequate tumor cell content of over 80% in most cases and at least 10% in regressive tumor samples was established from adjacent tissue sections. Whenever possible, we used DNA from blood samples and adjacent tumor-free kidney as controls.

Genetic alterations were identified through WES in 118 tumor and 78 control samples. WGS data were available for 31 cases (46 tumor, 41 control samples), and targeted Sanger sequencing was performed on 30 WT samples (22 stromal-type, 8 epithelial-type) suspected of *WT1* or *TRIM28* mutations. Additionally, copy number profiling and methylation-sensitive MLPA of the BWS imprinting control regions (BWS-IC1/2) were conducted to evaluate regions implicated in WT.

### Genetic and epigenetic WT predisposition

Across 129 children, we found a genetic predisposition in 73 and epigenetic predisposing events in 44 (Fig. 1B, Additional file 1: Table S2, S3). *WT1* alterations were the most common germline genetic driver. 27% of patients (35 patients) had truncating or missense mutations, or structural alterations in the *WT1* gene already in control tissue. *TRIM28* was the second most frequent genetic driver, altered in 9% of cases (12 patients, 2 being monozygotic twins). Additional affected genes previously described as potential WT drivers were *REST* (6 patients), *DIS3L2* (5 patients), *CTR9*, *DICER1*, *CDC73*, and *NONO* (1 patient each). Familial cases were among those with *WT1, TRIM28, REST, DIS3L2, CTR9,* and *CDC73* drivers.

Ten patients had germline mutations affecting cancer predisposition genes not typically linked to WT: *CHEK2* in 4 children, and *BLM*, *BRCA2*, *CDKN2A*, *FMN2*, *PIK3C3*, and *STK11* in one patient each. Another patient had Edwards syndrome (mosaic trisomy 18), a condition with increased risk for hepatoblastoma and WT. Except for the mosaic *NONO* mutation and homozygous *DIS3L2*,* BLM*, and *BRCA2* alterations in syndromic patients (Perlman, Bloom, Fanconi anemia), all predisposing genetic changes occurred as heterozygous germline events.

Analysis of the BWS imprinting region on chromosome 11p15.5 revealed a high frequency of epigenetic predisposing events. We found methylation alterations in blood or normal kidney tissue in 34% (44/129) of children. These children lacked other constitutional driver mutations. In contrast to germline mutations, these imprinting alterations often occurred as early somatic events, reflected by a mosaic loss of imprinting (LOI) or loss of heterozygosity (LOH) in normal kidney tissue or variable presence in blood cells. Isolated hypermethylation of BWS-IC1 (*IGF2*/*H19* region) was the most common alteration, seen in 38 patients. Another 5 patients exhibited mosaic LOH, characterized by a biallelic paternal methylation status with hypermethylation of IC1 and hypomethylation of IC2. One patient had partial hypomethylation of only IC2 in blood DNA. There were no familial cases that were epigenetically driven.

### *WT1* predisposition

*WT1* mutations are the most frequent genetic cause of WT predisposition, and they are linked to subsequent WNT activation. Among the 35 patients with germline *WT1* alterations, there was a gender imbalance (13 female, 20 male), and two patients were XY females (1 DDS, 1 non-syndromic). In one family, maternal inheritance is known (patient CIF). In the second, inheritance is unknown, but 11p LOH with a completely paternal *IGF2*/*H19* methylation pattern suggests paternal inheritance.

**Fig. 2:**
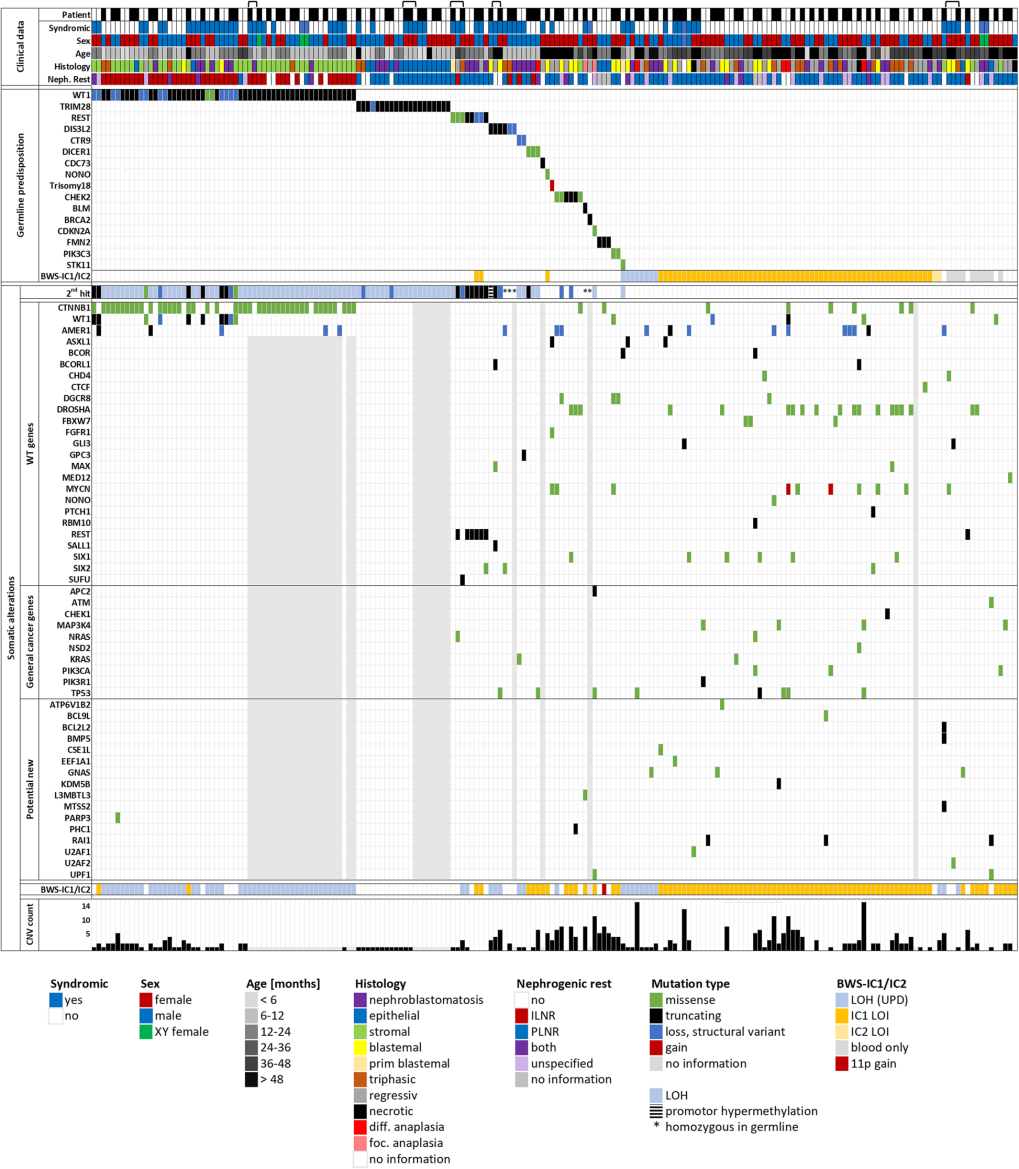
Clinical features and molecular alterations of bilateral and familial WT cases. Each column represents one tumor sample. Patients are indicated by alternating black and white boxes in the top row; adjacent boxes of the same color denote bilateral and/or relapse tumors. Familial cases are joined by brackets. 2^nd^ hit denotes the mechanism of complete inactivation of the predisposing gene. Details can be found in Additional file 1: Tables S2, S3 and Additional file 2: Fig. S2

All 35 germline *WT1* variants are predicted to be deleterious to function: 28 truncating variants (14 nonsense, 12 frameshift, and 2 splice site mutations), 1 hotspot missense mutation (D464G), and 6 large WAGR or limited *WT1* exon deletions or chromosomal rearrangements (Fig. 2, and Additional file 2: Fig. S1). Complete loss of WT1 function in tumors was due to somatic copy-neutral LOH of 11p in 43/53 samples, while 10 were compound heterozygous with 6 truncating mutations, 2 missense substitution or in-frame insertion variants, and 2 deletions of C-terminal exons (exons 6–10 or 7–10). These compound heterozygous alterations occurred preferentially in tumors with large (WAGR) or small structural *WT1* variants that may prevent a reduction to homozygosity. The second *WT1* hit occurred independently on both sides in all 11 informative bilateral cases, with the LOH region differing in 6 cases and distinct somatic SNVs in 5 cases (Additional file 1: Table S4).

*WT1* driver mutations were associated with somatic *CTNNB1* mutations in 41/53 tumors. Most of these affected the phosphorylation sites of the GSK3-mediated phosphodegron in exon 3 (*n* = 37), while 4 tumors had C-terminal alterations affecting the armadillo repeats (K335I, W383G, W383R, N387K). Of the 12 samples that lacked *CTNNB1* mutations, 3 had *AMER1* loss-of-function alterations instead. In a single case of *WT1*/*CTNNB1* mutant primary tumors, the local relapse only carried the *WT1* Y295* mutation without apparent WNT activation, but additional 1q gain and 16q loss as WT progression markers. Eight out of the 9 remaining *WT1*-driven samples had been classified as nephroblastomatosis, with neither *CTNNB1* nor *AMER1* mutations. In addition, WNT-activating mutations were also absent from tumor-associated nephrogenic rests (Additional file 1: Table S4) supporting the stepwise progression from nephrogenic rest to WT first proposed by Fukuzawa et al. [33].

**Fig. 3:**
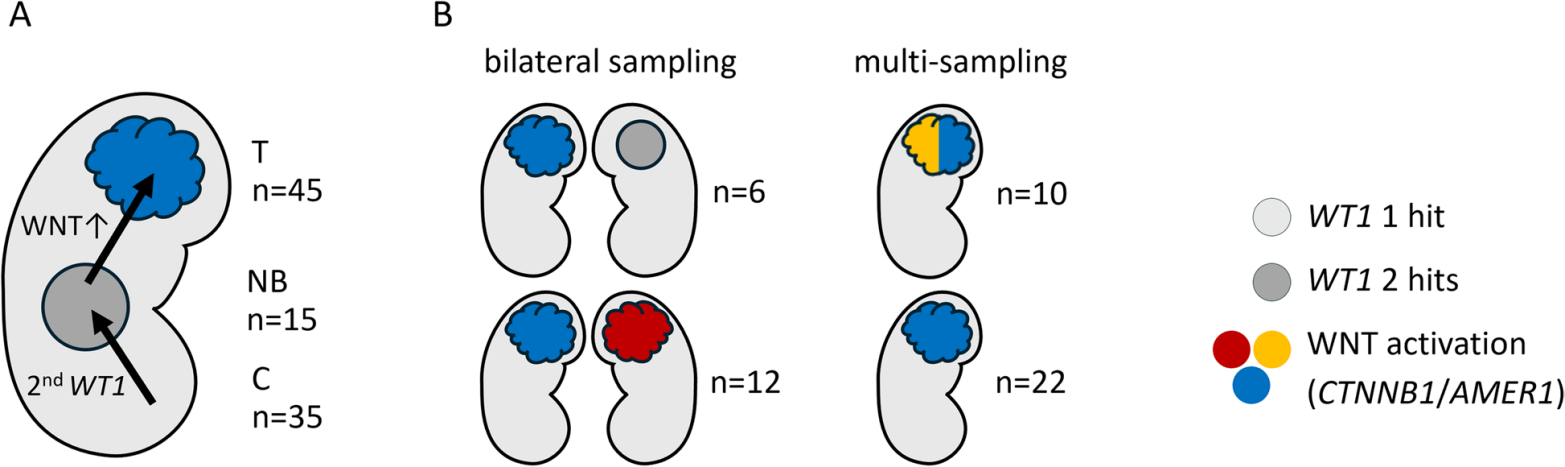
Somatic WNT activating mutations in *WT1-*driven tumors. **A** While the kidney parenchyma (C) was heterozygous for the *WT1* mutation, nephroblastomatosis/nephrogenic rest samples (NB) had an additional *WT1* mutation or became homozygous through 11p LOH. Only full tumors (T) carried *CTNNB1* and/or *AMER1* mutations. **B** When material was available from both sides, the second *WT1* hit in contralateral samples occurred independently (each of 11 informative cases). In 12 cases with tumors from both sides, contralateral tumors harbored different WNT activating alterations. Multiple biopsies (≥ 3) were available for 32 WT and these carried different *CTNNB1/AMER1* mutations within 10 of these tumors

Somatic WNT activation is an independent progression step that can occur multiple times (Fig. 3, Additional file 1: Table S4). In each of 12 cases in which tumors were available from both sides, *CTNNB1* and/or *AMER1* mutations were different. In 10/32 tumors with ≥ 3 separate ipsilateral samples, we likewise detected more than one *CTNNB1/AMER1* mutation indicative of originally multicentric WT (Fig. 3).

WES or WGS were available for 20/35 patients with *WT1* predisposition. Interestingly, none of the other known WT genes was affected in these tumors. Furthermore, the frequency of CNVs was very low, with LOH 11p, affecting both *WT1* and BWS-IC1/IC2, being the main alteration (Additional file 2: Fig. S2, and summarized in Fig. 5A).

### *TRIM28* predisposition

The second most frequent predisposition gene is *TRIM28* that encodes a transcriptional regulator with multiple roles in development and suppression of endogenous retroviral transcription [34]. Twelve children harbored a pathogenic *TRIM28* germline mutation, with equal gender distribution. Three children had a family history of WT (PCU, GKB, AQP) with maternal inheritance, and two were monozygotic twins (CDU + XDA) (previously reported in [35], see Additional file 1: Table S2). Germline events were primarily truncating *TRIM28* mutations (nonsense, indel or splice site), and in one child (PCU) the first three exons were deleted (Fig. 2). The majority of the 20 tumors analyzed had copy-neutral LOH 19q as a somatic event. In two cases, *TRIM28* inactivation was due to secondary deletion of a small (60–70 Mb) telomeric region of 19q.

*TRIM28*-driven tumors were genetically uniform based on WES/WGS analysis in 7 patients (12 tumors): besides a somatic loss of *TRIM28* function, there were no other oncogenic drivers or chromosomal aberrations, and unaltered BWS-IC1/2 imprinting (Fig. 5A). Surprisingly, there was no genetic difference between morphologically malignant epithelial tumors and neoplastic tissues exclusively consisting of nephroblastomatosis.

### Other WT genes

Germline variants in *REST*, a transcriptional repressor with important functions in differentiation and embryonic development [36], were detected in six patients. Three were siblings from one family, while the others had bilateral WT, but no other affected family members. One child with a non-familial WT (ZOZ) had BWS (mosaic *IGF2 *LOI in the kidney) in addition to a pathogenic heterozygous *REST* germline mutation. The spectrum of mutations included deletion of the whole *REST* gene, missense, nonsense, and frameshift variants. Except for one tumor with copy-neutral LOH 4q including the mutant *REST* locus, all other tumors had independent second somatic *REST* mutations.

*DIS3L2* alterations were detected in 2 families (3 patients) and in 2 patients with Perlman syndrome. *DIS3L2* encodes an exoribonuclease that plays a crucial role in WT tumorigenesis, especially via the let7-Lin28 pathway [37]. The three familial patients had germline heterozygous deletions of exon 9. In the tumor, the second allele was inactivated by independent deletions of exons 9 or 13–21, or by promoter hypermethylation with transcriptional silencing. The patients with Perlman syndrome had homozygous germline mutations - either frameshift or deletion of exons 6–13.

Singular cases of familial predisposing mutations were: A patient with a heterozygous deletion of *CTR9* exons 8–9, that became homozygous due to LOH 11p in the left and right tumor. In this family, the father and two children had Wilms tumor. One patient (DKS) had a *CDC73* truncating (Y293*fs) germline variant that was inherited from the father, while another sister carries the mutation and has no WT at the age of 17.

We found a heterozygous germline *DICER1* G803E mutation in one child without a clinical diagnosis of DICER1 syndrome. *DICER1* encodes an endoribonuclease involved in miRNA processing. The G803E mutation affects an evolutionarily conserved amino acid residue and a similar G803R mutation has been described in a large family where 2 of 11 mutation carriers developed WT, and 4 of 11 individuals with germline G803R had other phenotypes of DICER1 syndrome [38]. The child (LWW) had bilateral nephroblastomatosis which later progressed to unilateral WT. The nephroblastomatosis lesions carried an additional somatic frameshift mutation in *DICER1*, or a somatic copy-neutral LOH including the *DICER1* locus. The latter progressed to a diffuse anaplastic WT with *TP53* R175H mutation.

The *NONO* R75H hot spot mutation appeared as a mosaic mutation in a girl (ZJN): a small fraction of normal kidney cells (AF 0.03, 2/76 reads) already had the alteration that became heterozygous in the tumor DNA (AF 0.57). Sequencing of tumor-derived cDNA revealed monoallelic expression of the NONO R75H mutation, indicating that the active X-chromosome was affected.

In general, tumors driven by germline WT gene alterations showed bi-allelic inactivation of the driver gene, by either copy-neutral LOH, loss of the remaining wildtype allele, an independent somatic event, or promoter hypermethylation with gene silencing. In 5 of 19 tumors, additional known WT genes (*AMER1*,* SIX2*,* MAX*,* SALL1*,* SUFU*,* GPC3*) were altered, and 4 tumors had additional mutations in general cancer genes (*TP53, NRAS, KRAS*). There was also a higher number of CNVs in this subgroup (Additional file 2: Fig. S2).

### General cancer predisposition genes

In 10/129 patients, we found germline variants in general cancer predisposition genes including *CHEK2*, *BLM*, *BRCA2*, *CDKN2A*, *STK11*, and candidate cancer predisposition genes, *FMN2* and *PIK3C3*. Four patients harbored heterozygous *CHEK2* germline alterations, R562Q and I157T and twice T367Mfs*15. The latter two are common in the population (AF 0.3% and 0.5%), but they appear to confer an increased risk for multiple cancers, e.g., breast and prostate cancer [39, 40]. The remaining wild-type allele of *CHEK2* was lost through LOH 22q in 2/6 tumors.

A homozygous *BLM* truncation was detected in a patient (QFA) with signs of dysmorphia of unknown origin at the time of diagnosis. The mutation is consistent with the phenotype of Bloom syndrome, which confers a high risk to develop different types of cancer including WT at an early age [41]. Biallelic germline *BRCA2* truncating mutations were found in one familial case (WYE), where 2/3 children developed WT, but there was no tumor material to analyze somatic alterations.

Germline variants not previously reported in WT were: *CDKN2A* R24Q (XJN) and *STK11* L245F (QXN), both of which became homozygous in the tumor through LOH. The germline *CDKN2A* R24Q variant is associated with familial melanoma [42]. The patient with the *STK11* mutation later developed adrenocortical carcinoma, followed by a diagnosis of Peutz-Jeghers syndrome 11 years later.

An intriguing heterozygous *FMN2* nonsense mutation was detected in blood DNA of a patient with bilateral WT (QVX). FMN2 is proposed to control spindle positioning and chromosome segregation, especially in meiosis, and loss of function leads to polyploidy [43]. Trisomies 7, 9, 12, and 18 were present in both primary tumors and as a subclonal event in normal kidney (20%), which indicates early (embryonic) occurrence of these trisomies. Trisomies 12 and 18 are frequent somatic alterations in WT and may represent the initial tumor drivers.

A heterozygous PIK3C3 missense mutation (R642H) was observed in one non-syndromic patient (WCN). PIK3C3 is a regulator of autophagy and, in addition, could induce oncogenic transformation and enhance tumor cell proliferation, growth, and invasion through mechanisms independent of autophagy [44]. The mutation affects a conserved residue within the catalytic domain of PIK3C3 and is predicted to be pathogenic by common prediction tools. It has been reported as a somatic mutation in the Cosmic database in various cancer types.

Tumors initially driven by general cancer genes depend on additional somatic mutations. These were found in 10/14 tumors, affecting WT genes (e.g. *DGCR8*, *DROSHA*, *MYCN*) and genes with known oncogenic properties, like *APC2*, *UPF1*, *PHC1*, and *L3MBTL3* (Fig. 2, summarized in Fig. 5A). Chromosomal copy number alterations were much more frequent with a mean of 4.7 (0–10) events (Additional file 2: Fig. S2).

### Epigenetic predisposition

Forty-four of the 56 patients for whom no predisposing DNA sequence alteration could be identified showed epigenetic WT predisposition. Forty-three patients exhibited complete or often mosaic hypermethylation of BWS-IC1 (*H19*/*IGF2* region) in healthy control tissues: 38 cases with at least partial LOI at IC1 in kidney or blood and 5 cases with LOH 11p15 affecting both IC1 and IC2. In one case, hypomethylation of IC2 was seen - a common cause of BWS that seems to be very rare in WT, however [45]. IC1 imprinting alterations leading to biallelic *IGF2* expression are frequent events in WT, and they are associated with BWS when present in constitutional DNA. However, only six of our patients had been diagnosed with symptoms of overgrowth (4 BWS, 2 hemihypertrophy).

Tumors based on epigenetic predisposition are likely dependent on additional, secondary somatic driver mutations (Figs. 2 and 5A). These include several WT genes like miRNA processing genes, *MYCN*, *SIX1/2*, and *CTNNB1/AMER1*, as well as known cancer genes, e.g., *TP53*, *MAP3K4*, and *PIK3CA*. In addition, we found mutations in several genes not yet described as recurrently affected in WT, but with known oncogenic properties in other cancer types (*BCL9L*, *CSE1L*, *EEF1A1*, *GNAS*, *KDM5B*, *RAI1)*. These may merit further investigation in future cohort studies. There was an intermediate level of CNVs (0–14, mean 2.5) in these tumors, suggesting a strong need for additional oncogenic changes.

### Mosaic imprinting defects

**Fig. 4:**
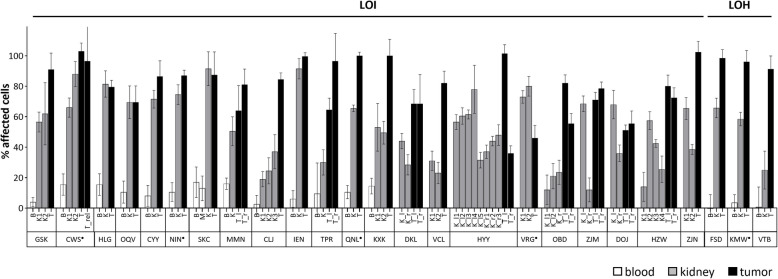
Heterogeneity of early somatic BWS-IC1 methylation alteration. Methylation status was determined in blood (B, white bar), muscle (M, white), multiple normal kidney biopsies (K, gray) and tumor (T, black) samples by MS-MLPA. The percentage of affected cells is given based on the mean of 4 probes (LOI) or 8 probes (LOH) of the MS-MLPA kit. Patients CWS, VRG, and QNL have been diagnosed with Beckwith-Wiedemann spectrum disorder, and NIN and KMW with hemihypertrophy (labeled with •), while neither condition has been reported for all other patients

To further study the mosaic nature of imprinting defects, we analyzed multiple control samples (blood and/or kidney) from 25 patients, with paired blood and kidney DNA available for 16 of them. Blood samples often showed little or no evidence of imprinting defects, with a maximum of 20% affected cells, consistent with the rare diagnosis of BWS features (Fig. 4). On the other hand, corresponding kidney samples had a much higher proportion of cells with imprinting defects, ranging from 10% to almost complete hypermethylation (median 50%). In all six cases with material available from both kidneys, *IGF2* imprinting defects were detected in both, indicating an early origin, before the separation of precursors leading to left and right kidney primordia. The low levels of imprinting defects in blood DNA suggest that several of the 12 unresolved cases - 7 with LOI in the tumor - may result from epigenetic predisposition that could not be verified due to the lack of normal kidney controls.

### Relapse and progression

Tissues from six local relapses at 6–35 months and from one late metastatic event were available for study. The local relapses appeared to represent independent metachronous Wilms tumors as they shared the predisposing mutation, but only a limited number of further progression events (Additional file 1: Table S2). In *WT1*-driven tumors, the extent of LOH corresponded to the ipsilateral primary tumor. Thus, it seems likely that smaller dormant lesions remained in the kidney after nephron-sparing surgery and subsequently progressed to full malignancy, fueled by an independent set of secondary mutations. The subsequent lung metastasis from a child with WT1 predisposition differed from the right primary tumor in the extent of the LOH 11p, suggesting that it was derived from the left primary tumor, which was not available for study. The sometimes higher number of genetic alterations or the TP53 mutation in one of the recurrences is consistent with the corresponding older age.

### Syndromic associations

**Fig. 5:**
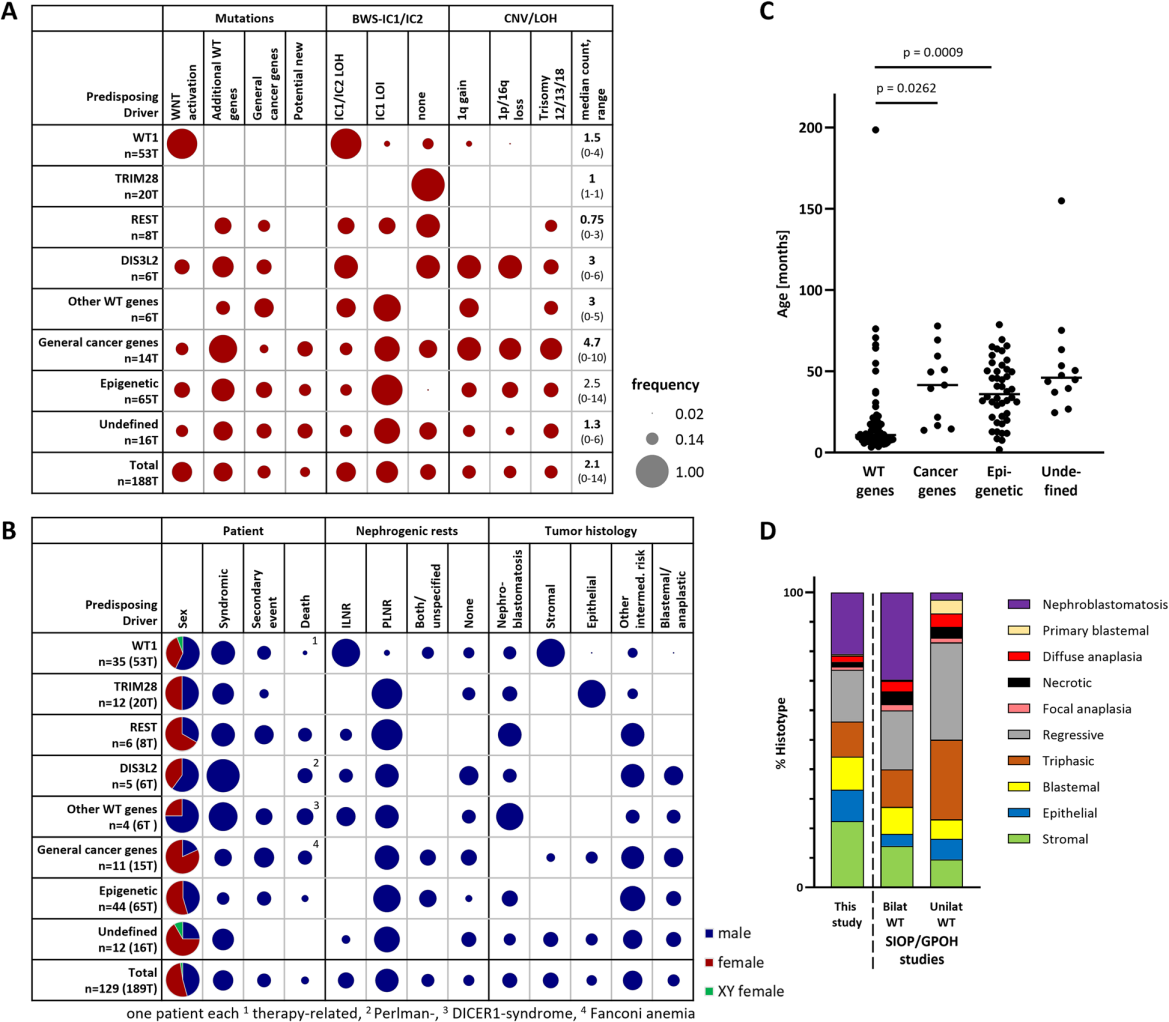
Associations between clinical and molecular findings. **A** Somatic alterations in different predisposition subgroups. **B** Clinical characteristics and histopathological findings in relation to the predisposing driver mutations. Numbers denote patients, with the total number of tumors listed in brackets. Syndromic features include family history of WT, clinically defined syndromes (DDS, WAGR, Perlman, Edwards, BWS, hemihypertrophy), urogenital malformation, and cryptorchidism. **C** Age at diagnosis listed as per class of driver mutation. **D** Distribution of histological WT subtypes in this study and in bilateral vs. unilateral WT cases in the SIOP/GPOH database

Predisposition features had been described at diagnosis in 36% (47/129) of the patients (Fig. 5B, Additional file 1: Table S2). This included family history (20 patients), clinically defined syndromes (DDS, WAGR, Perlman, Edwards, BWS, hemihypertrophy; 20 patients), urogenital malformation and cryptorchidism (6 patients), or unclassified features (1 patient).

Syndromes or a corresponding clinical phenotype had been reported for 14/32 sporadic *WT1-*driven cases. Five patients diagnosed with Denys-Drash syndrome harbored typical exon 8/9 missense (D464G) or nonsense mutations (R430*, R458*), but also N-terminal truncating mutations (M309fs*1, A123fs*69). Unexpectedly, 5 additional patients carried the same constitutional exon 8/9 nonsense mutations (1 × R430*, 4 × R458*), but lacked an initial diagnosis of DDS. Three patients with WAGR syndrome had large germline deletions at 11p13 including *WT1*. Isolated genitourinary malformations were seen in 6 male patients with truncating *WT1* mutations. Most of these patients presented with synchronous bilateral tumors, but three had metachronous bilateral disease (intervals of 4.3 to 7 years), and one (QLH) developed a WT1-based secondary AML (acute myeloid leukemia).

No syndromic features were reported in the *TRIM28*-based cases, besides familial overgrowth in one patient (KAR). In patients with *REST*, *CTR9*, and *CDC73* germ-line mutations, there were likewise no clinical signs other than family history. While homozygous *DIS3L2* and *BLM* mutations came to attention early on through the development of corresponding syndromes, *BRCA2*, *DICER1*, and *STK11*-associated syndromes were only diagnosed after the initial WT diagnosis. None of the patients with other cancer predisposition gene mutations showed clinical abnormalities. Only 14% of patients (6/44) with epigenetic predisposition presented with clinical evidence (BWS, hemihypertrophy) of the underlying defect.

### Clinical and histopathological characteristics

Tumors in children with typical WT gene drivers were diagnosed at a younger age than epigenetically predisposed tumors or those with general cancer predisposition mutations (Fig. 5C). In bilateral disease, nephroblastomatosis is diagnosed far more frequently than in unilateral cases (29.5% vs. 2.5%), which is also reflected in our cohort (21%) (Fig. 5B, 5D).

In general, *WT1*-driven tumors had stromal predominant histology (38/53) or were classified as nephroblastomatosis (9/53). They carried nephrogenic rests in 89% of the cases, 72% being intralobar, 4% perilobar, and 13% both or unspecified. In contrast, the majority of *TRIM28* mutant tumors had epithelial histology (14/20) or were classified as nephroblastomatosis (4/20), and all but one had perilobar nephrogenic rests. Tumors based on other WT genes or general cancer predisposition mutations and *IGF2* imprinting defects were histologically more diverse: stromal or epithelial histology was rare, but triphasic or regressive tumors were frequent (43%) and there was a high rate of high-risk histology (21%).

In total, 23/129 (18%) patients developed a secondary event, and eight patients died - one from therapy, five from disease progression, and two from underlying syndromes. While patient HDU with Perlman syndrome died due to multi-organ involvement, patient WYE with Fanconi anemia suffered from a second malignancy (glioblastoma/anaplastic oligodendroglioma).

## Discussion

Our cohort represents the largest collection of consecutively collected bilateral and familial WT cases reported to date, allowing the contribution of different predisposition mechanisms to Wilms tumorigenesis to be estimated. In 91% (117/129) of cases, we determined the underlying predisposing alteration present in the germline or in early somatic cells, exceeding prior success rates. More than half of the tumors (73/129) were driven by genetic DNA sequence alterations, and another third (44/129) had (mosaic) epigenetic predisposition to WT formation. Most of the affected genes were recessive, requiring a second somatic hit.

The largest subgroup of 35 cases was due to a *WT1*-based predisposition. There was a striking escalation pattern from predisposed kidneys with heterozygous *WT1* mutations to nephrogenic rests or nephroblastomatosis exhibiting complete *WT1* inactivation together with two paternal imprints on BWS-IC1, while full conversion to a malignant WT seems to rely on additional activation of WNT signaling. The latter occurred mostly via *CTNNB1* exon 3 mutations that inactivate a phosphodegron motif (83%). In a smaller number of cases, mutations of the *CTNNB1* armadillo repeats or inactivation of *AMER1*, which is part of the ß-catenin destruction complex, were found. This is consistent with earlier reports on sporadic tumors with nephrogenic rests, where WNT activation in *WT1*-driven cases was limited to tumor tissue but absent from nephrogenic rests [33]. *AMER1* mutations occurred together with *CTNNB1* mutations, but only the less frequent type affecting the Arm repeats, not the phosphodegron variants. This same pattern is seen in data from the TARGET analysis [46] and it may be related to the differential potency of CTNNB1 Arm repeat vs. phosphodegron mutations [47].

The WNT activating mutations often differed between sides and even within one side in presumably multifocal tumors, providing further evidence of a stepwise progression from precursor lesions to WT. The paucity of other secondary driver mutations and the low incidence of copy number alterations fit well to the picture of a rather stereotypic development of these tumors: (1) the initial heterozygous germline *WT1* mutations in all kidney cells, (2) complete *WT1* inactivation and concomitant upregulation of *IGF2* expression via two paternal IC1 imprints, and (3) WNT activation via *CTNNB1* and/or *AMER1*.

*TRIM28*-mediated predisposition appears to act even more simplistic with biallelic mutations in both nephrogenic rests and tumor samples as the only genetic change, but the determinants of progression from nephrogenic rest to WT remain unresolved [35]. All other predisposing mutations were rare, and the spectrum of affected genes was limited compared to sporadic WT. A similar restriction in the number of potential drivers was seen in the COG study where 25/61 cases could be attributed to a predisposing mutation, with *WT1* (N=9), *NYNRIN* (N=4), and *TRIM28* (N=3) being affected most frequently [48]. This reduced spectrum may be due to some of the typical WT driver mutations/genes being incompatible with normal kidney development even in the heterozygous state, only allowing for late somatic inactivation that would not lead to bilaterality.

The number of epigenetic alterations on chromosome 11p15 as the predisposing event was surprisingly high (34%), but only a minority of these patients (6/44) were clinically known to have BWS or hemihypertrophy. This is consistent with the frequent mosaicism observed in BWS that poses a challenge to genetic testing [49]. In 43/44 of epigenetically predisposed cases, BWS-IC1 was affected. In a meta-analysis of 1370 epigenotyped patients with BWS, only 0.1% (1/836) with isolated IC2 hypomethylation developed WT, whereas 6.2% (21/341) with paternal uniparental disomy (pUPD) affecting IC1 and IC2, and 21.1% (26/123) with IC1 hypermethylation had WT [15]. This clearly identifies IC1 as the most relevant imprinting center for WT predisposition, which supports our findings.

In our bilateral cases, imprinting defects must have occurred as early somatic mosaics in mesodermal cells whose descendants still contribute to both sides of the body. The lack or limited presence of imprinting defects in blood samples is consistent with a time point after the separation of hematopoietic and metanephric cell lineages that may vary between patients. It is thus possible that some of the 12 cases that lacked evidence of a predisposing alteration, but showed LOI in tumor cells, may likewise originate via imprinting defects. However, the lack of a normal kidney sample for confirmation, or a random low abundance of cells with altered imprinting may have precluded detection.

A similar variability has been seen in a smaller series of WTs, where clonal expansions in kidney precursors were characterized, carrying methylation changes at the IC1 region [50]. Likewise, alterations at the IC1 locus - mostly hypermethylation - have been reported in lymphocyte DNA of 3% (13/437) of non-syndromic WT patients with sporadic tumors that lacked features of growth disorders [51]. These data argue for a continuum from a > 20% WT risk in BWS with IC1 hypermethylation to increasingly lower risks with mosaic IC1 imprinting defects, depending on their time of occurrence and grade of mosaicism.

The frequent mosaic IC1 imprinting alterations indicate that not only tumors, but also adjacent kidney samples should be tested for such changes, in order to detect mosaic individuals. There is no correlation between the reporting of a clinical diagnosis of BWS or hemihypertrophy and the proportion of affected blood or normal kidney cells, and in most cases, there was no prior evidence for syndromic features. Therefore, one cannot rely on prior syndrome diagnostics. Tumors may, in turn, inform on the occult presence of a mosaic status, which can then be investigated further.

An important outcome of this study is that a large fraction of children with predisposition can be identified even in technically less equipped countries. This is especially true for stromal and epithelial tumors and those with epigenetic predisposition that together encompass more than 2/3 of cases. Here, a cost-effective approach of histopathology, or immunohistochemistry followed by targeted sequencing, or a simple MLPA-based test of imprinting will be highly informative, similar to the escalating analysis done for some of the samples described in this study: *WT1*-mutant bilateral tumors exhibit stromal morphology, which informs genetic testing. While boys with germline *WT1* mutation frequently present with urogenital malformation and cryptorchidism, girls are phenotypically normal and *WT1* germline mutations are easily overlooked. Thus, one may consider determining the *WT1* status of all stromal tumors (in particular if intralobar nephrogenic rests are present) and, if mutated, to test germline DNA to identify patients at risk and their siblings at an early stage. Some patients with WT1 mutations go on to develop *WT1*-driven AML later in life that could also be amenable to surveillance.

Epithelial WT should likewise be tested for *TRIM28* mutations as this is the second most prevalent mutation in bilateral cases. Our prior work has demonstrated that 50% of all epithelial WT carry such mutations, with half of them being present in germline DNA without syndromic features [35]. Such cases with risks for patients and siblings might otherwise be overlooked.

The restricted number of genes that may carry predisposing variants facilitates the development of a targeted panel, which will allow for efficient screening of affected children. Given that more than 10% of cases are bilateral or familial and that there are presumably additional unilateral cases with predisposing mutations, general genetic screening of WT appears to be a worthwhile endeavor. This is even more true because some of the predisposing variants may carry a risk for other cancers or diseases later in life, examples being mutations in *BRCA2*, *STK11*, *BLM*, and *CDKN2A*, among other genes. Germline *WT1* mutations often lead to impaired kidney function in later life. Here in particular, liquid biopsy testing in parallel to preoperative chemotherapy can indicate such risks and lead to a preference for nephron-sparing surgery in order to preserve the functional renal parenchyma as far as possible. For all other cases with predisposing mutations, the possibility of metachronous tumors may likewise favor a more conservative surgical approach.

The number of patients with genetic predisposition may be underestimated in our study, as unilateral multifocal tumors are difficult to identify and demarcate, and they are often analyzed just once. Especially, the stereotypic progression in *WT1-*mutant tumors permits clear identification of multiple independent transforming events. Thus, multifocality, as illustrated by distinct WNT activation events in our series of multi-sampled tumors, may have gone unnoticed in prior studies.

Weaker or less penetrant predisposing alleles may also only lead to unilateral disease that is not covered by our approach. Thus, the fraction of tumors arising in predisposed children will be higher than previous estimates of around 10%, in line with the report by Hol et al. [7]. This all argues in favor of combining tumor plus germline genetic analyses in Wilms tumor patients, if not already covered by liquid biopsy. A drawback is the frequent lack of family information, also in our study. It is therefore difficult at present to estimate penetrance and thus, actual risks for siblings or descendants, but heightened awareness and the broad application of molecular diagnostics should help to alleviate this problem in the future.

## Conclusions

Our study shows that there are two prevailing mechanisms of predisposition to bilateral WT: either germline genetic alterations acting through mutant *WT1*, *TRIM28*, and a small number of additional WT and general cancer genes; or to a slightly lesser extent, postzygotic mosaic imprinting defects of BWS-IC1, which controls *IGF2* expression. While *WT1-* and *TRIM28-*based oncogenesis follows a strict and predictable pattern, the other predisposition genes and epigenetic alterations use more diverse pathways to achieve the full tumorigenic state. They also use a wider range of secondary drivers and chromosomal alterations. Taken together, our data provide a clear path for future molecular diagnostics of Wilms tumor predisposition that can be directly implemented in treatment protocols. The high proportion of genetic tumor predisposition strongly supports liquid biopsy screening at diagnosis. Besides tumor risk classification, it can provide convincing arguments for nephron-sparing surgery to maximize renal tissue preservation, given the risk of metachronous contralateral disease or syndromic limitations in renal function.

## Supplementary Information

Supplementary Material 1. Table S1. Primers for targeted analysis. Table S2. Clinical and molecular data of patients and corresponding tumors. Table S3. Genomic position (hg19) of sequence alterations. Table S4. WNT activating mutations in WT1-driven tumors.

Supplementary Material 2. Fig. S1: Germline and somatic *WT1* alterations in *WT1*-driven tumors. Fig. S2. Clinical features and molecular alterations of bilateral and familial WT cases.

## Data Availability

All data supporting the findings of this study are available within the manuscript and its Supplementary Information. Complete human sequence data generated in this study are not publicly available due to patient privacy requirements but are available upon reasonable request sent to the corresponding author (manfred.gessler@uni-wuerzburg.de). Applications will be decided at the latest at the biannual meeting of the study commission.

## Abbreviations

AML: Acute myeloid leukemia
BWS: Beckwith-Wiedemann syndrome
DDS: Denys-Drash syndrome
GPOH: Gesellschaft für Pädiatrische Onkologie und Hämatologie
IC1/2: Imprinting center 1/2
LOH: Loss of heterozygosity
LOI: Loss of imprinting
MLPA: Multiplex ligation-dependent probe amplification
SIOP: International Society of Paediatric Oncology
WAGR: Wilms tumor, aniridia, genitourinary anomalies and range of developmental delays
WES: Whole exome sequencing
WGS: Whole genome sequencing
WT: Wilms tumor
