# Supplementary material for: Distinct pathways for genetic and epigenetic predisposition in familial and bilateral Wilms tumor
Source: Genome Med. 2025 May 8;17:49. doi: 10.1186/s13073-025-01482-0 (PMC12060375; doi:10.1186/s13073-025-01482-0)
Supplement: Supplementary file 2 — Supplementary Material 2. Fig. S1: Germline and somatic WT1 alterations in WT1-driven tumors. Fig. S2. Clinical features and molecular alterations of bilateral and familial WT cases. [file 13073_2025_1482_MOESM2_ESM.pdf]

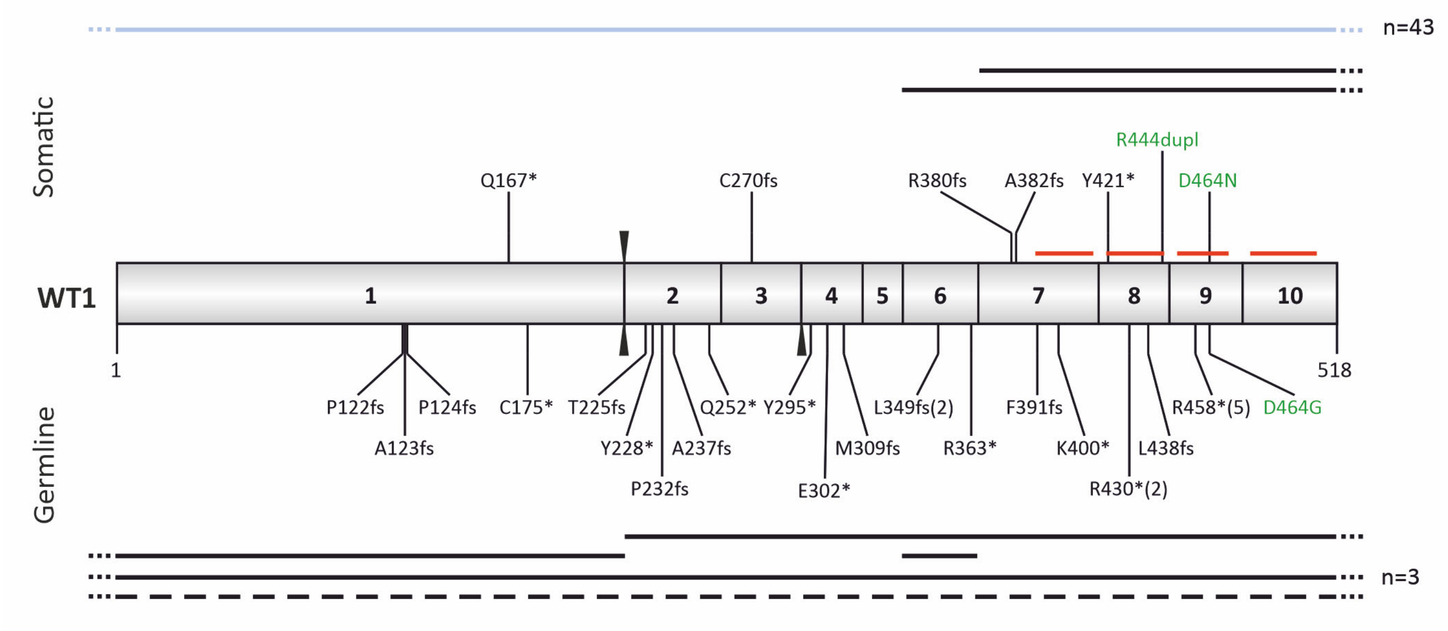

Fig. S1: Germline and somatic *WT1* alterations in *WT1*-driven tumors. The exon structure (exons 1-10) of *WT1* is shown, with red bars indicating Zn-finger coding regions. Germline (lower panel) and somatic (upper panel) alterations are indicated with respective amino acid position. ▲(splice site mutation), black solid line (deletion), dashed line (rearrangement), or blue line (LOH) indicate larger changes. Truncating variants are labeled in black, missense mutations in green. The number of tumors affected is shown in brackets.

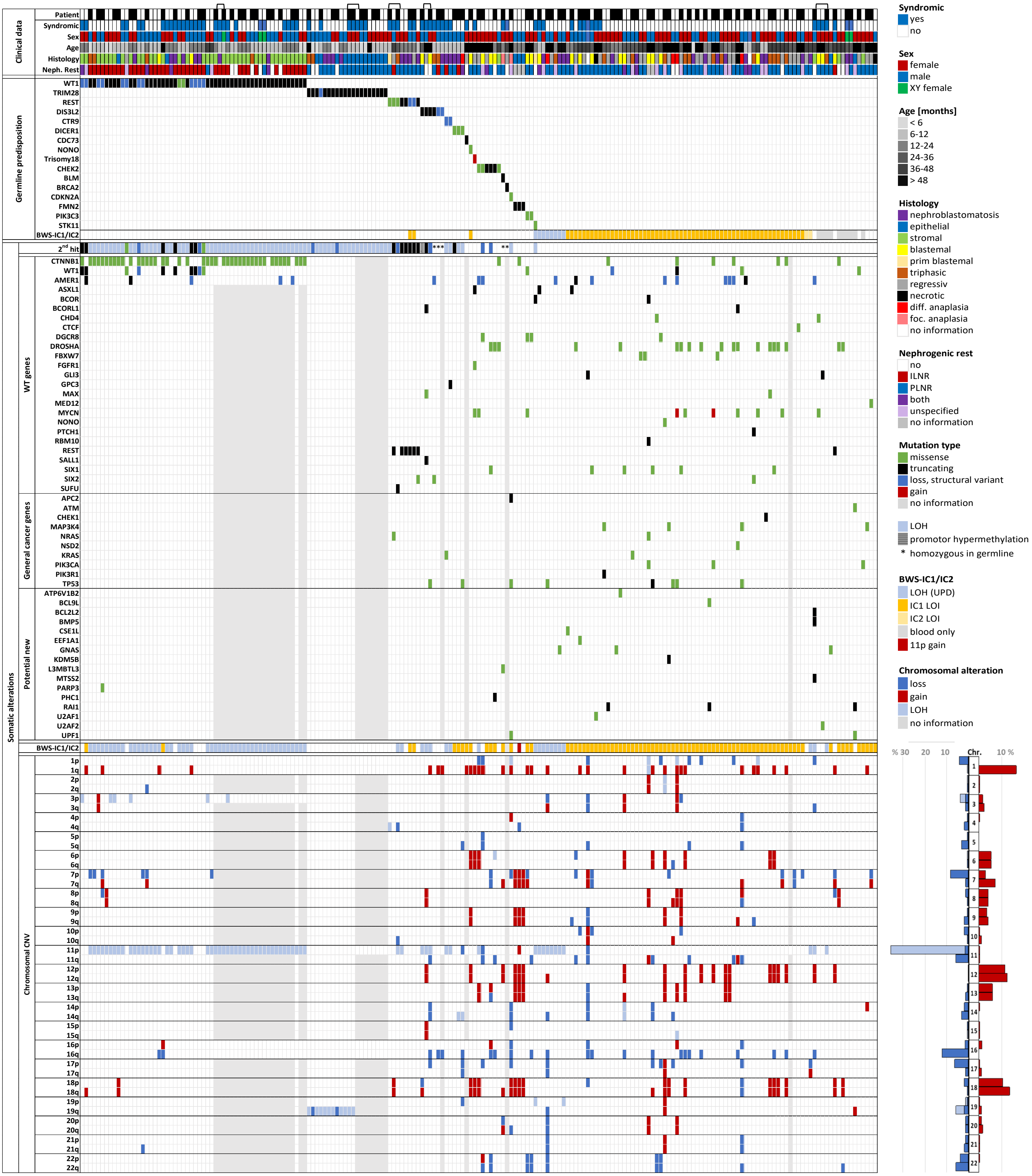

Fig. S2: Clinical features and molecular alterations of bilateral and familial WT cases. Each column represents one tumor sample. Patients are indicated by alternating black and white boxes in the top row, adjacent boxes of the same color denote bilateral and/or relapse tumors. Familial cases are joined by brackets. 2nd hit denotes the mechanism of complete inactivation of the predisposing gene. Sequence alterations as well as chromosomal alterations are given for individual tumors as listed in Additional file 1: Table S2. Relative frequency of LOH, gain or loss of the respective chromosomal arms in the analyzed tumor cohort is shown at the bottom right
